# Deciding on genetic testing for familial dementia: Perspectives of patients and families

**DOI:** 10.1002/alz.70140

**Published:** 2025-04-06

**Authors:** Jetske van der Schaar, Sven J. van der Lee, Eva C. A. Asscher, Yolande A. L. Pijnenburg, Christa M. de Geus, Annelien L. Bredenoord, Wiesje M. van der Flier, Mariette A. van den Hoven, Ellen M. A. Smets, Leonie N. C. Visser

**Affiliations:** ^1^ Section Genomics of Neurodegenerative Diseases and Aging Department of Human Genetics Vrije Universiteit Amsterdam Amsterdam Netherlands; ^2^ Alzheimer Center Amsterdam Neurology, Vrije Universiteit Amsterdam Amsterdam UMC location VUmc Amsterdam The Netherlands; ^3^ Amsterdam Neuroscience, Neurodegeneration Research & Diagnostics Center (RDC) ‐ ADORE Amsterdam The Netherlands; ^4^ Department of Ethics Law and Humanities Amsterdam Netherlands; ^5^ Clinical Genetics Department of Human Genetics Vrije Universiteit Amsterdam Amsterdam Netherlands; ^6^ Erasmus School of Philosophy Erasmus University Rotterdam Rotterdam The Netherlands; ^7^ Department of Epidemiology & Data sciences Vrije Universiteit Amsterdam Amsterdam UMC location VUmc Amsterdam The Netherlands; ^8^ Department of Medical Psychology Amsterdam UMC location AMC Amsterdam The Netherlands; ^9^ Amsterdam Public Health Quality of Care Amsterdam The Netherlands; ^10^ Department of Bioethics and Health Humanities Julius Center for Health Sciences and Primary Care University Medical Center Utrecht Utrecht University Utrecht The Netherlands

**Keywords:** Alzheimer's disease, considerations, decision‐making, DNA‐diagnostics, familial dementia, frontotemporal dementia, genetic testing, interest, Lewy body dementia, monogenic causes, pathogenic mutations, patient perspective, predictors, symptomatic testing, vascular dementia

## Abstract

**INTRODUCTION:**

We explored patients’ and families’ interest in, predictors of, and considerations regarding genetic testing for monogenic causes of dementia in a diagnostic setting.

**METHODS:**

This mixed‐methods study evaluated 519 consecutive Alzheimer Center Amsterdam patients for monogenic testing eligibility. Among those qualifying, differences between testers and non‐testers were analyzed. Thirty‐three patients completed questionnaires. Additionally, we conducted 21 semi‐structured interviews with 15 patients and 18 relatives. Verbatim transcripts were analyzed inductively.

**RESULTS:**

Of 138 (27%) eligible patients (46% female, age 61 ± 8 years, Mini‐Mental State Examination [MMSE] 22 ± 6), 75 (54%) underwent genetic testing. Testers had better cognition, higher quality of life, and more often undetermined diagnoses than non‐testers (all *p *< 0.05). Decisions were guided by intuitive, value‐driven judgments: testers sought to provide heredity information to relatives, enhance actionability, and reduce uncertainty, while non‐testers worried about psychosocial impact on family, or unfavorable timing.

**DISCUSSION:**

The substantial interest in genetic testing for monogenic causes of dementia underscores the need for further research into the implications of disclosing test results to memory clinic patients.

**Highlights:**

- Half of memory clinic patients’ who met eligibility criteria proceeded with genetic testing.
- Those tested were more likely to have an undetermined diagnosis, better cognition, and higher quality of life.
- Decisions were motivated less by deliberation of factual information, and more by quick, intuitive judgments.
- Motivations pro included providing information, enhancing actionability, and resolving uncertainty.
- Motivations con comprised concerns about the emotional burden and disruptive impact on their family.

## 1 BACKGROUND

Dementia is characterized by progressive cognitive decline, affecting multiple domains, such as memory, language, and executive function, thereby impairing daily functioning.^1^ This syndrome can result from various pathologies, including Alzheimer's disease (AD), vascular dementia (VaD), dementia with Lewy bodies (DLB), and frontotemporal dementia (FTD).^2^ The vast majority of cases are considered multifactorial, resulting from a complex interplay of genetic, environmental, and lifestyle factors.^3^ However, in a minority of instances, dementia originates from a pathogenic mutation in a single disease‐associated gene, for example, *PSEN1* (causing early‐onset AD), *C9ORF72* (causing FTD and amyotrophic lateral sclerosis [ALS]), or *NOTCH3* (causing Cerebral Autosomal Dominant Arteriopathy with Subcortical Infarcts and Leukoencephalopathy [CADASIL], which leads to VaD).^4^, ^5^ These monogenic causes often manifest before the age of 65, with some patients presenting symptoms in their fifties, forties, or even thirties. As inheritance patterns are mostly autosomal dominant, children of an affected parent have 50% risk of carrying a genetic abnormality, and if so, are very likely to develop the disease due to penetrance ranging from high to complete.^6^, ^7^

Over the past few decades, monogenic causes of dementias have been identified in more than 50 genes.^8^, ^9^, ^10^, ^11^ Advances in next‐generation sequencing technologies, and in particular the introduction of diagnostic panels to simultaneously examine multiple genes, have facilitated genetic testing as part of the diagnostic work‐up in clinical practice.^12^ Yet, historically, clinicians have been reluctant to test dementia patients for monogenic causes due to ethical concerns. These include the limited clinical utility in the absence of therapeutic interventions, compounded by the profound psychological and societal impacts on patients and their families when confronted with their genetic predisposition to dementia. Serious adverse effects can comprise anxiety, depression, suicidal ideation, as well as stigmatization and discrimination, for example, in employment and insurance contexts.^13^ However, when used appropriately, genetic testing can provide diagnostic clarity, for example, by distinguishing neurodegenerative from psychiatric conditions, thereby avoiding harmful delays in diagnosis. This affords patients and their families time to organize care and support, prepare for disease progression, or participate in clinical trials.^9^ In addition, insight in the cause may reduce uncertainty, improve understanding, and enables at‐risk relatives to consider predictive testing or make informed decisions about life choices, for example, family planning.^14^, ^15^

Recent scientific progress is expanding medical utility and personal actionability for patients and at‐risk relatives. Due to advances in the development of disease‐modifying treatments, more opportunities to enroll in clinical trials designed to delay or defer neurodegeneration are becoming available for carriers of high‐penetrance mutations for familial AD^16^ and FTD.^17^ Additionally, innovations in reproductive technologies, such as in vitro fertilization with preimplantation genetic diagnosis, now offer the possibility of preventing the transmission of these hereditary conditions to future generations.^18^ These considerations for potentially at‐risk relatives may play a role in evaluating whether to pursue genetic testing for symptomatic patients.

While a substantial body of literature covers genetic counseling and testing in families with an established inherited disorder like Huntington's disease (HD),^19^, ^20^, ^21^ less attention has been given to diagnostic testing within routine care, which may reveal hereditary dementia in previously unrecognized or unsuspecting families. As the latter becomes increasingly available in memory clinics, comprehensive guidelines and recommendations are essential. We previously developed data‐driven criteria to assess eligibility of symptomatic patients for testing for monogenic causes.^8^ The perspectives of patients and their families on the possibility of genetic testing as part of the tertiary diagnostic work‐up are crucial for optimal implementation of genetic counseling and testing, yet they remain largely unknown. To bridge this gap, we evaluated consecutive patients at our memory clinic over a 1‐year period, and if they met our eligibility criteria, offered them monogenic testing to explore: (1) their interest; (2) demographic, medical, and psychosocial predictors of interest in genetic testing; and (3) their considerations regarding the decision to (not) be tested for monogenic causes of dementia.

## 2 METHODS

### 2.1 Study design

This observational study employed a mixed‐methods approach to investigate perspectives of patients and their family members on testing for monogenic causes of dementia. The protocol was approved by the by the Ethics Committee of Amsterdam UMC (#2021.0534) on October 1, 2021. All methods were carried out in accordance with relevant guidelines and regulations.

**RESEARCH IN CONTEXT**

1. **Systematic review**: The authors reviewed the literature using traditional databases (e.g., PubMed). Publications on the perspectives of patients and families regarding symptomatic testing of potential probands for monogenic causes of dementia in a memory clinic setting are scarce. Most studies focused on other neurodegenerative diseases, particularly amyotrophic lateral sclerosis (ALS) and Parkinson's disease (PD). These are cited as appropriate.
2. **Interpretation**: Our findings are in line with previous reports on decision‐making regarding monogenic testing for ALS and PD, suggesting a considerable proportion of patients who meet eligibility criteria are interested to learn whether dementia has a genetic cause, and consider this information to be personally and medically actionable.
3. **Future directions**: Longitudinal studies are needed to corroborate present results on decision‐making, and explore the psychosocial and behavioral implications of disclosing tests results. Given the recent advances in developing disease‐modifying therapies and growing opportunities to participate in prevention trials, it is essential to take patients’ and families’ perspectives into account.

This research was conducted at a tertiary memory clinic, with a high proportion of relatively young patients with complex clinical presentations. The vast majority (> 95%) provide informed consent for the use of their clinical data for research purposes.^22^, ^23^

**FIGURE 1:**
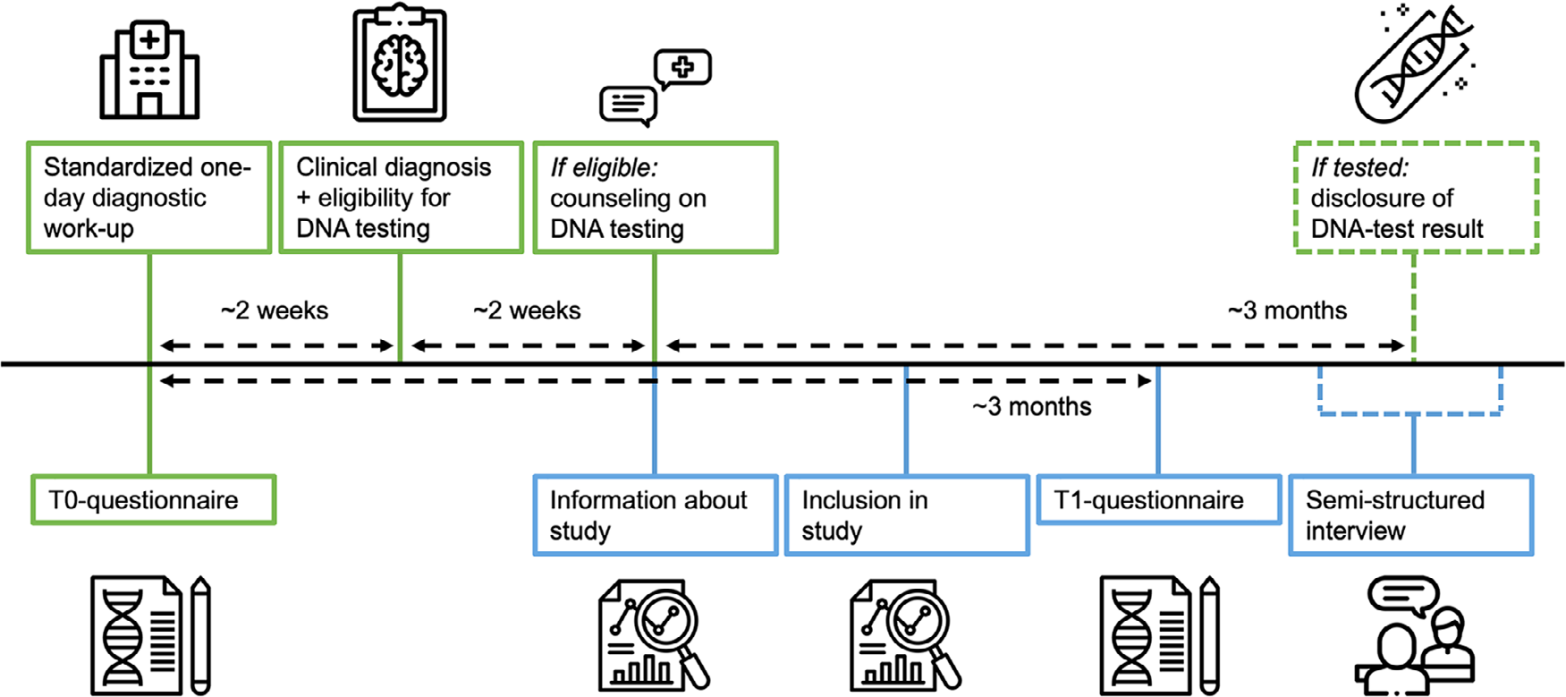
Timeline of patient visits and assessments. Green blocks represent procedures of standard care in Alzheimer Center Amsterdam, blue blocks represent procedures of the study regarding decision‐making factors. The T0‐questionnaire measures the following psychosocial factors: Perceived susceptibility for, severity of, and experience with dementia, assessed by first two subscales and an added third of the Motivation to Change Lifestyle and Health Behaviors for Dementia Risk Reduction Scale (MCLHB‐DRR)^24^, ^25^; Perceived likelihood of genetic cause, assessed by visual analog scale (VAS); Openness to discuss symptoms in the family, assessed by adapted version of Openness to Discuss Cancer in the Family (ODCF) scale^26^, ^27^; Perceived social support, assessed by Multidimensional Scale of Perceived Social Support (MSPSS)^28^; Coping strategies, assessed by BRIEF‐COPE scale^29^; Anxiety and depression, assessed by Hospital Anxiety and Depression Scale (HADS)^30^; Quality of life, assessed by Brunnsviken Brief Quality of Life Scale (BBQ)^31^ and VAS. The T1‐questionnaire measures the following decision‐making factors: Beliefs; Knowledge of genetic causes; Knowledge of heredity risk; Perceived likelihood of genetic cause, assessed by VAS; Considerations; Shared decision‐making. Constructs in T1‐questionnaire are assessed by self‐developed scales, drawing from literature on factors potentially associated with interest in and impact of decision‐making in (pre‐)symptomatic testing for genetically inherited disorders, as validated scales were not available. Full versions of T0‐ and T1‐questionnaires are provided in Supplement 1.

As shown in Figure 1, at their first visit (T0) patients underwent a standardized one‐day diagnostic work‐up consisting of medical, neurological, and neuropsychological assessment, magnetic resonance imaging (MRI) and optional cerebrospinal fluid (CSF) analysis. In addition, they filled out a questionnaire assessing psychosocial factors potentially predicting patients’ interest to (not) proceed with genetic testing, that is, perceived susceptibility, perceived severity, and experience with dementia, perceived likelihood of a genetic cause, openness to discuss symptoms in the family, perceived social support, anxiety and depression, and quality of life (based on,^24^, ^25^, ^26^, ^27^, ^28^, ^29^, ^30^, ^31^ see Supplement 1). Demographic and medical data were retrieved from patients’ records.

In a multi‐disciplinary meeting, the clinical diagnosis was established. Based on this, age at presentation and family history in first‐degree relatives, patients were evaluated using decision criteria (see Supplement 2) to determine eligibility for genetic testing.^8^ The latter comprised (1) pathogenic genetic variants (Class IV and V based on the American College of Medical Genetics and Genomics)^32^ in a 54‐gene dementia panel (targeted exome‐sequencing), (2) the *C9ORF72* hexanucleotide repeat expansion,^33^ and (3) amyloid precursor protein (*APP*) duplications.^34^ A detailed description of the genetic panel is reported elsewhere.^8^ The treating neurologist shared the suspected diagnosis with patients and their caregivers. Those who met the criteria were offered information about diagnostic testing for monogenic causes of their symptoms, and referred for additional counseling by a geneticist in case of highly suspicious family history.

If eligible for genetic testing, at least 18 years old and able to fill out questionnaires (optionally supported by a caregiver), the treating physician invited patient and/or one or more relatives for inclusion in the study, regardless of the decision to proceed with testing or not. After written informed consent was obtained, participants filled out a T1‐questionnaire, assessing considerations regarding the decision to (not) proceed with genetic testing (see Figure 1 and Supplement 1).

To further explore their perspectives on genetic testing, patients and family members were invited to participate in optional semi‐structured in‐depth interviews. These lasted 45–60 min, were conducted with either the patient, a family member or both, and took place at home, digitally via Teams or in person at Alzheimer Center Amsterdam, depending on the participants’ preference. Topics included motivations to engage in or refrain from genetic testing, considerations relevant to their decision, and potential implications of learning the results (see Supplement 3 for topic guide).

### 2.2 Quantitative analysis

All fully answered scales were included in analysis, regardless of whether the entire questionnaire was completed. Differences in demographic, medical, and psychosocial factors between patients who decided in favor or against genetic testing were calculated using Pearson's χ^2^, Mann‐Whitney *U*, or independent samples *t*‐tests; *p*‐values < 0.05 were considered significant. Due to the exploratory nature of our aims, no adjustment for multiple comparisons were performed. All analyses were performed using the statistical software R version 4.3.2 (R Foundation for Statistical Computing).

### 2.3 Qualitative analysis

Jetske van der Schaar conducted all interviews and audio recordings were transcribed verbatim. We followed an inductive approach to allow concepts to emerge directly from the participants' narratives.^35^ Jetske van der Schaar and Eva C. A. Asscher examined the content to generate descriptive codes, which were then iteratively refined and organized into broader categories, to capture the richness and complexity of perspectives. The resulting coding tree was discussed in regular meetings between Jetske van der Schaar and Eva C. A. Asscher, and subsequently used to code all interviews by Jetske van der Schaar. To enhance the reliability and validity, three interviews were double coded by Jetske van der Schaar and Eva C. A. Asscher. Discrepancies were discussed, and consensus was reached when agreement was achieved on the majority of codes applied to the same segments. Main themes were formulated by Jetske van der Schaar based on the frequency, coherence, and relevance of codes across the dataset, to capture the most salient aspects of participants' perspectives, and reviewed and refined in multiple rounds with input from Eva C. A. Asscher, Wiesje van der Flier, and Leonie Visser. Data were managed using MAXQDA‐software version 22.1.1. Results are reported in accordance with the Consolidated Criteria for Reporting Qualitative Research (COREQ) guidelines.^36^

## 3 RESULTS

**FIGURE 2:**
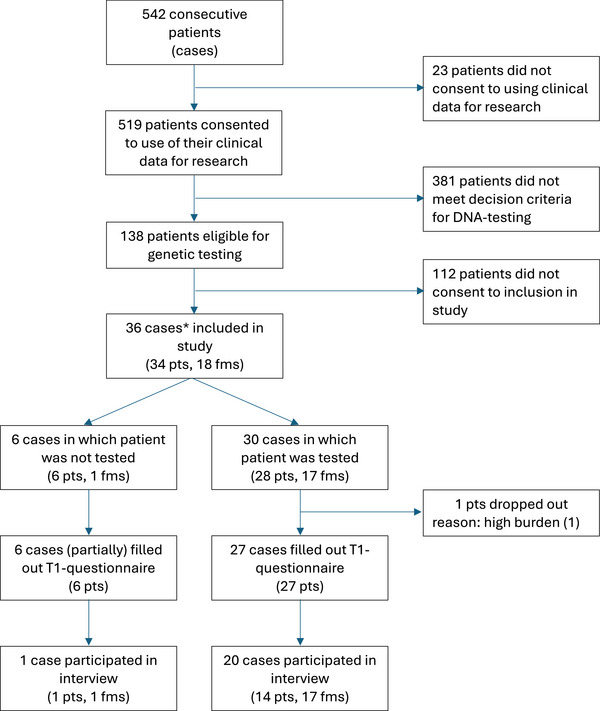
Flow diagram of participants. ^*^A case refers to a potential proband, that is, an individual eligible for genetic testing for monogenic causes of dementia. For each case, either the patient, one or more family members, or both were included in the study (specifically, in five cases only patients were included, in two only family members, and in the remaining cases both). Only patients were offered genetic testing, family members were invited to participate in optional interviews. fms, family members; pts, patients

As shown in Figure 2, between October 18, 2021, and October 26, 2022, 542 consecutive patients had their first visit and 519/542 (96%) consented to the use of their clinical data for research purposes. Of these, 496 filled out the T0‐questionnaire. After evaluating all for eligibility, 138/519 (27%) were offered genetic testing.

### 3.1 Patients’ interest in genetic testing

The demographic, clinical, and psychosocial characteristics of the patients eligible for genetic testing (*n *= 138) are shown in Table 1. Nearly half were female (64/138, 46%) and average age at presentation was relatively young (61 ± 8 years), in line with the typical early‐onset of familial dementia. Half self‐reported a family history (68/138, 49%) and the majority had children (110/138, 80%). Their perceived likelihood of a genetic cause for their symptoms was 37% (sd = 31%).

**TABLE 1:** Demographic, medical, and psychosocial characteristics of all eligible patients, stratified by the decision to proceed with genetic testing (no/yes)

Parameter	All (*n* = 138)	No (*n* = 63)	Yes (*n* = 75)	*p*‐Value
Demographic characteristics				
Sex [*n* (%) female]	64 (46%)	32 (51%)	32 (43%)	0.34
Age (mean±sd years)	61±8	61±7	61±9	0.85
Education (mean±sd)	5±1	5±1	5±1	0.90
Has children [*n* (% yes)]	110 (80%)	48 (76%)	62 (83%)	0.35
Geographical origin [*n* (%) European, Moroccan, Turkish, other, not reported]	100 (72%)	49 (78%)	51 (68%)	0.66
	2 (1%)	0 (0%)	2 (3%)	
	4 (3%)	2 (3%)	2 (3%)	
	8 (6%)	3 (5%)	5 (7%)	
	24 (17%)	9 (14%)	17 (20%)	
Clinical characteristics				
Has family history [*n* (% yes)]	68 (49%)	31 (49%)	37 (49%)	1
MMSE (mean ± sd)	22±6	21±6	23±5	0.04^*^
Diagnosis				0.01^*^
SCD [*n* (%)]	5 (4%)	1 (2%)	4 (5%)	
MCI [*n* (%)]	9 (7%)	5 (8%)	4 (5%)	
Dementia [*n* (%)]	98 (71%)	50 (79%)	48 (63%)	
AD (*n*)	64	39	25	
FTD (*n*)	12	3	9	
VaD (*N*)	3	1	2	
DLB	1	0	1	
PPA	11	6	5	
Other (*n*)	7	1	6	
Other/undetermined [*n* (%)]	26 (19%)	7 (11%)	19 (29%)	
Psychosocial characteristics				
Perceived susceptibility for dementia (3–15) (mean±sd)	10±3	10±3	10±3	0.34
Perceived severity of dementia (5–25) (mean±sd)	16±4	16±4	15±4	0.52
Experience with dementia (2–10) (mean±sd)	6±3	6±3	6±2	0.61
Perceived likelihood of genetic cause (%±sd)	37±31	34±30	39±32	0.42
Openness to discuss symptoms in family (9–45) (mean±sd)	33±6	34±6	33±6	0.70
Perceived social support (1–7) (mean±sd)	6±1	5.9±0.9	5.9±1.0	0.63
Coping strategy				
Problem‐focused (1–4) (mean±sd)	2.6±0.8	2.6±0.7	2.5±0.8	0.69
Emotion‐focused (1–4) (mean±sd)	2.2±0.5	2.2±0.5	2.3±0.6	0.36
Avoidant (1–4) (mean±sd)	1.8±0.6	2.0±0.5	1.9±0.6	0.07
Anxiety (0–21) (mean±sd)	6.5±4.7	7.0±4.8	6.2±4.5	0.44
Depression (0–21) (mean±sd)	5.3±4.3	6.0±4.7	4.9±4.0	0.32
Quality of life				
BBQ (0–96) (mean±sd)	54±22	53±22	54±21	0.74
VAS (1–10) (mean±sd)	7.1±1.8	6.7±2.0	7.5±1.6	0.02^*^

Abbreviations: AD, Alzheimer's disease; BBQ, Brunnsviken Brief Quality of Life Scale; DLB, dementia with Lewy bodies; FTD, frontotemporal dementia; MCI, mild cognitive impairment; MMSE, Mini‐Mental State Examination; PPA, primary progressive aphasia; SCD, subjective cognitive complaints; sd, standard deviation; VaD, vascular dementia; VAS, visual analog scale.

* We found group differences in MMSE, diagnosis distribution and quality of life as assessed by a VAS (*p* < 0.05).

Education was rated using the Dutch Verhage system (range: 1‐7). Other instruments are listed in Figure 1, a detailed description of the specifics is reported in Supplement 1.

Comparison between patients’ decisions were calculated with Pearson's χ^2^, Mann‐Whitney *U*, or independent samples *t*‐tests.

Of those eligible, just over half (75/138, 54%) decided to proceed with genetic testing, while a slightly smaller proportion refrained from doing so. In 54/75 (72%) cases, test results were negative, in 14/75 (19%), a cause was found in one gene (*AARS2*, *AMACR*, *CTSF*,^2^ *GRN*, *HTRA1*, *MAPT*, *NOTCH3*,^2^ *PSEN1*,^3^ *SPG7*) or a triplication encompassing multiple genes (16q24.2q24.3), and in 6/75 (8%), variants of uncertain significance were identified. One was pending when we last assessed clinical records for this study.

### 3.2 Factors associated with interest in genetic testing

Table 1 shows the demographic, clinical, and psychosocial characteristics for the 138 eligible patients, stratified by the decision to proceed with genetic testing (no / yes). When comparing groups, differences in MMSE (21 ± 6 vs. 23 ± 5, *p* = 0.04), distribution of diagnoses (*p* = 0.01), and quality of life as assessed by a visual analog scale (VAS) (6.7 ± 2.0 vs. 7.5 ± 1.6, *p* = 0.02) were observed, indicating that better cognition, an undetermined diagnosis, and higher quality of life were associated with the decision to proceed with genetic testing. No other demographic, medical, or psychosocial factors were related to the decision to proceed with genetic testing.

### 3.3 Considerations

Thirty‐four patients and 18 family members related to 36 cases participated in the study (in 5 cases only patients were included, in two only family members, and in the remaining cases both; 30 cases decided in favor of and six against genetic testing; Figure 2). Of these, 33 patients (partially) filled out the T1‐questionnaire, meaning they completed at least one, but not necessarily all scales. The demographic, clinical, and psychosocial characteristics of these patients are shown in Table 2. Descriptive statistics of the results are reported in Supplement 4. In addition, we conducted 21 interviews (three with patients, six with family members, and 12 with both). Nine took place before and 12 after disclosing test results. Fifteen cases tested negative, four positive; in one, a variant of uncertain significance was found, and one refrained from testing.

**TABLE 2:** Demographic, medical, and psychosocial characteristics of all eligible patients who filled out the T1‐questionnaire, stratified by the decision to proceed with genetic testing (no/yes)

Parameter	All (*n* = 33)	No (*n* = 6)	Yes (*n* = 27)	*p*‐Value
Demographic characteristics				
Sex [*n* (%) female]	13 (39%)	1 (17%)	12 (44%)	0.36
Age (mean±sd years)	61±7	60±4	61±8	0.72
Education (mean±sd)	5±1	5±1	5±1	0.75
Has children [*n* (% yes)]	29 (88%)	6 (100%)	23 (85%)	1
Geographical origin [*n* (%) European, Moroccan, Turkish, other, not reported]	26 (79%)	5 (83%)	21 (78%)	1.00
	0 (0%)	0 (0%)	0 (0%)	
	0 (0%)	0 (0%)	0 (0%)	
	1 (3%)	0 (0%)	1 (4%)	
	6 (18%)	1 (17%)	5 (19%)	
Clinical characteristics				
Has family history [*n* (% yes)]	18 (55%)	3 (50%)	15 (56%)	1
MMSE (mean±sd)	23±5	19±3	24±5	0.05
Diagnosis				0.96
SCD [*n* (%)]	2 (6%)	0 (0%)	2 (7%)	
MCI [*n* (%)]	1 (3%)	0 (0%)	1 (4%)	
Dementia [*n* (%)]	25 (76%)	5 (83%)	20 (74%)	
AD (*n*)	17	5	12	
FTD (*n*)	0	0	0	
VaD (*N*)	1	0	1	
DLB	1	0	1	
PPA	4	0	4	
Other (*n*)	2	0	2	
Other/undetermined [*n* (%)]	5 (15%)	1 (17%)	4 (15%)	
Psychosocial characteristics				
Perceived susceptibility for dementia (3–15) (mean±sd)	10±3	9±2	10±3	0.23
Perceived severity of dementia (5–25) (mean±sd)	16±5	16±3	16±5	0.97
Experience with dementia (2–10) (mean±sd)	6±3	6±3	6±2	0.68
Perceived risk of genetic cause (%±sd)	38±35	13±12	44±36	< 0.05^*^
Openness to discuss symptoms in family (9–45) (mean±sd)	35±5	36±3	35±5	0.66
Perceived social support (1–7) (mean±sd)	6±1	6.3±0.4	6.2±0.7	0.68
Coping strategy				
Problem‐focused (1–4) (mean±sd)	2.6±0.7	2.5±0.6	2.6±0.7	0.61
Emotion‐focused (1–4) (mean±sd)	2.3±0.6	1.9±0.6	2.4±0.5	< 0.05^*^
Avoidant (1–4) (mean±sd)	1.8±0.5	1.6±0.3	1.8±0.5	0.39
Anxiety (0–21) (mean±sd)	6.5±4.8	4.2±3.0	7.0±5.0	0.19
Depression (0–21) (mean±sd)	4.4±3.5	4.3±3.0	4.4±3.7	0.96
Quality of life				
BBQ (0–96) (mean±sd)	53±18	49±15	54±19	0.55
VAS (1–10) (mean±sd)	7.5±1.4	7.3±1.1	7.5±1.4	0.68

Abbreviations: AD, Alzheimer's disease; BBQ, Brunnsviken Brief Quality of Life Scale; DLB, dementia with Lewy bodies; FTD, frontotemporal dementia; MCI, mild cognitive impairment; MMSE, Mini‐Mental State Examination; PPA, primary progressive aphasia; SCD, subjective cognitive complaints; sd, standard deviation; VaD, vascular dementia; VAS, visual analog scale.

* We found group differences in perceived likelihood of genetic cause and emotion‐focused coping strategy (*p* < 0.05).

Education was rated using the Dutch Verhage system (range: 1–7). Other instruments are listed in Figure 1; a detailed description of the specifics is reported in Supplement 1.

Comparison between patients’ decisions were calculated with Pearson's χ^2^, Mann‐Whitney *U*, or independent samples *t*‐tests.

The main findings from the qualitative thematic analysis were organized into three main categories: *knowledge and perceptions (section 3.3.1)*, *decision‐making strategies (3.3.2)*, and *motivations (3.3.3)*. For each, we report the key themes that emerged from the interviews, illustrated with quotes from participants and supplemented by findings from the questionnaires.

#### 3.3.1 Knowledge and perceptions

##### Limited knowledge of monogenic causes

Qualitative and quantitative data revealed that knowledge of monogenic causes of dementia was limited. In interviews, many participants referred to the genetic variants they were tested for as risk factors, assuming mutations do not necessarily lead to dementia: “If it's hereditary, of course, the question remains whether you will definitely have to deal with it” (family member, case 110011, tested). Most participants were unaware of potential implications of learning they carried such a cause, including financial discrimination, stating: “I haven't worried about that, I assume regulations are fine” (patient, case 110039, tested). Questionnaires further showed that patients perceived their likelihood of a single inherited factor to explain their symptoms at 34% (sd = 33%), with those pursuing testing feeling at greater risk than those opting out (41% vs. 8%, *p* < 0.05). The chance of first‐degree relatives to share the familial predisposition was estimated at 43% (correct answer: 50%), and if so, the risk for them to develop dementia was at 49% (correct answer: depending on the penetrance of the mutation, up to 100%). Furthermore, most patients (24/33, 73%) did not realize a genetic cause could lead to increased insurance premiums.

##### Challenges in knowledge uptake

In interviews, participants described several challenges in their uptake of knowledge of monogenic causes, including the overwhelming period in the disease trajectory (“If you've just received the diagnosis, and someone explains very well how [genetic testing] works, it doesn't really sink in”; family member, case 110028, tested), the affected cognition of the patient (“It's just a lot of information, and I think it's challenging in any case, especially for [my partner], to remember it all”; family member, case 110010, tested), as well as the pressure of time to make an informed decision (“You shouldn't wait too long because it becomes increasingly difficult for people with Alzheimer's to discuss these matters effectively”; family member, case 110018, not tested). The timing of genetic testing also emerged as a notable consideration from the questionnaires, as patients who chose to proceed with genetic testing more often concurred that the timing was right (21/27, 78%), compared to those opting out (3/6, 50%).

#### 3.3.2 Decision‐making strategies

##### Intuitive and stepwise decision‐making approach

Interviews provided insight in participants’ decision‐making strategies. Interestingly, patients and family members mentioned that the knowledge of genetic risk and deliberation of potential implications was not essential for determining their course of action: “I actually feel like I shouldn't want to know everything, when scenarios are not yet in play” (family member, case 110010, tested). Rather than extensively weighing pros and cons, many participants reported that they quickly and intuitively made up their minds when offered the option of genetic testing: “We knew right away” (patient, case 110009, tested). Some had considered it beforehand, while others decided in the moment, attributing their decisions to character and personal values: “We are always very open with our children, in everything, so I believe they have the right to know whether it is [hereditary],” (family member, case 110019, tested). Many preferred to follow a stepwise approach: focusing on the current decision, awaiting the outcome, and considering future consequences only if and when they arise, thereby accepting the risk of unforeseen ramifications: “Sometimes reality is just very harsh. It is what it is” (patient, case 110012, tested).

##### Involvement of immediate family

From both qualitative and quantitative data, immediate family emerged as paramount in the decision‐making strategies. In interviews, most participants did not consider patients as the primary stakeholders in genetic testing: “Actually, it's really a question for the children” (family member, case 110010, tested). Many participants actively involved their sons and daughters, taking their interest and perspectives into account, whereas siblings were at best informed but did not have a say. One sister decided for the patient and took her to have blood drawn: “I think there was a bit of a selfish aspect involved. […] I want to know this for myself. […] I want to know this for my children” (family member, case 110035, tested). Questionnaires confirmed that most participants engaged in discussions with their families (27/33, 82%). Notably, compared to patients who proceeded with genetic testing, those opting out more often indicated having discussed the matter with their partners (6/6, 100% vs. 18/27, 67%), and more strongly endorsed being influenced by their relatives' opinions (5/6, 83% vs. 16/27, 59%).

#### 3.3.3 Motivations

##### Transparency about heredity

When invited to reflect on their motivations in interviews, participants emphasized that the primary relevance of genetic testing was “for my children and grandchildren, of course” (patient, case 110019, tested). Several mentioned a responsibility for parents to provide genetic information for the next and future generations: “If you suspect something, you must inform them, because they will be burdened by it eventually” (patient, case 110012, tested). Likewise, in questionnaires, patients strongly endorsed that their family should be aware of information about heredity (29/33, 88%). They considered it important to know the risk of dementia for their siblings or children (29/33, 88%), although those choosing to be tested agreed with this more than those who declined [26/27 (96%) vs. 3/6 (50%)].

##### Personal and medical actionability

Another key motivation emerging from the data was personal and medical actionability of genetic information. In interviews, participants focused on the importance of contributing to advances in scientific research. They believed this would yield clinical utility for their offspring, by equipping them with valuable information for family planning, a timely diagnosis, and preventive interventions: “At some point, one can say, you are at risk, but tomorrow or the day after, there might be a pill to slow it down or cure it or whatever. Then you catch it in time” (family member, case 110022, tested). In questionnaires, most patients affirmed that knowing the cause of their complaints would lead to better care and support (28/33, 85%). Furthermore, they expressed it would help them prepare for the time ahead by arranging care, advanced directives, and a will (28/33, 85%), while also enabling their relatives to make informed choices for the future (25/33, 76%).

##### Reducing uncertainty and gaining clarity

Another important motivation derived from the results was the value of understanding the origins of their disease. Several participants sought an explanation for what caused the dementia to develop at a young age, wondering if lifestyle might have played a role. Those who deemed the chance of a genetic cause to be relatively small, wanted to exclude the possibility and potential guilt of having passed on the mutation: “it's nice to know for yourself that it isn't hereditary for your children” (family member, case 110019, tested). Conversely, those who considered the risk to be substantial, wished to end the “psychological burden of the unknown. […] You may not want to know, but it's always on your mind, you'll still have that unease, so you might as well [get tested]” (family member, case 110039, tested). Likewise, in the questionnaires, many patients concurred they struggled with not knowing the origin of their symptoms, preferring to have clarity (24/33, 73%).

##### Emotional burden and disruptive impact on family

Lastly, another important motivation that surfaced from the data related to the psychosocial consequences of a positive test result, in particular the emotional burden of knowing relatives might be at risk, and the disruptive influence such knowledge could have on families. In interviews, participants elaborated on the “blow one would have to deal with” (family member, case 110009, tested), highlighting the grief and concerns this might bring: “I would, of course, find that terrible for [our children] and their future. And perhaps the fear they would then live with, that they could also develop a form of dementia at a young age” (family member, case 110015, tested). In questionnaires, two‐thirds of patients concurred that, if a genetic cause were found, they would worry about their relatives (5/6, 83%). Additionally, those who refrained from being tested more frequently indicated that such knowledge could strain relationships within their family compared to those who chose to proceed (3/6, 50% vs. 3/27, 11%).

## 4 DISCUSSION

4.1

In our mixed‐methods study, we found that half of memory clinic patients meeting eligibility criteria for genetic testing for monogenic causes of dementia chose to make use of this option. This decision was driven less by deliberation of factual information, of which participants demonstrated limited knowledge, and more by quick, intuitive judgments rooted in personal and family‐centered values. When asked about their motivations, those pursuing testing were driven by transparency, seeking to provide information about heredity to their families, enhancing actionability, facilitating informed decision‐making, and resolving uncertainty. Conversely, those opting out were concerned about their family's wellbeing, expressing that awareness of a genetic cause might lead to worry about their children, burden their relatives, and strain relationships, or felt that the timing was not right.

Over a 1‐year period, 27% of consecutive patients visiting Alzheimer Center Amsterdam met our data‐driven eligibility criteria.^8^ Of those, 54% opted to undergo genetic testing. Comparative data on uptake rates of symptomatic testing for monogenic causes of dementia in clinical settings are scarce. We found no other studies on this specific topic, and only two in other neurodegenerative diseases, particularly ALS^37^ and Parkinson's disease (PD).^38^ These disorders occur in both sporadic and familial forms, with the latter resulting from pathogenic mutations. Examples include hexanucleotide repeat expansions in the *C9ORF72* gene, which cause ALS as well as FTD,^39^ and *LRRK2* variants, which lead to PD and have also been linked to Lewy body dementia (LBD).^40^ A survey reported that 33% of patients with ALS had been offered genetic testing and 67% of them completed it,^37^ whereas a questionnaire among individuals with PD revealed that 59% desired to determine whether their condition resulted from a monogenic cause.^38^ These results align closely with our eligibility and interest rates.

Most patients who qualified for genetic testing presented at a tertiary memory clinic with symptoms of cognitive decline at a relatively young age (61 ± 8 years), and in 19% of cases, the etiology of their symptoms was unknown. Furthermore, 19% were found to carry a genetic cause, warranting further investigation into genetic origins in these individuals. We observed that patients who underwent testing were more likely to have an undetermined diagnosis, better cognition, and higher quality of life. These patients may have been more motivated to seek an explanation for their symptoms, better able to understand the purpose of genetic testing, and more psychologically resilient in managing the potential implications of the results.

Regarding the decision‐making process, both interviews and questionnaires revealed that participants’ knowledge of genetic testing for monogenic causes of dementia, and the potential psychosocial implications for patients and their families, was limited. This is consistent with a comparable study among patients with PD, reporting low levels of familiarity with heredity as well as an overestimation of their risk to carry a genetic cause.^38^ Specifically, 26% believed they had a genetic form of PD (although fewer than 5%–10% of cases do),^41^ whereas in our study, participants perceived the likelihood of having a monogenic cause of dementia to be 37% (while the prevalence in this memory clinic population is 3.3%^8^). This lack of comprehension of genetic risks has been a concern since the discovery of the gene marker for HD. Before predictive testing became available, 56%–81% of asymptomatic relatives of affected patients expressed a desire to know their genetic status, but since its clinical introduction, around 5%–20% have completed it.^13^, ^42^ Likewise, initial interest among individuals at risk for familial dementia was 50%–64%,^43^, ^44^, ^45^ whereas the actual uptake tends to remain at 5%–20%.^46^, ^47^, ^48^, ^49^ This substantial decline in willingness to be tested has been associated with a significant increase in knowledge, especially after a disease‐causing mutation has been identified in a relative.^50^

Patients and family members expressed that understanding genetic risks and overseeing potential implications were not crucial in determining their course of action. Instead, many participants made up their minds quickly and instinctively, attributing their decisions to character and personal values. These findings align with the Dual Process Model, which distinguishes two systems of thought that govern decision‐making.^51^ One is fast, automatic, and intuitive, relying on mental heuristics, whereas the second is slow, deliberate, and analytical, demanding more cognitive resources. Participants in our study appeared to predominantly and intentionally resort to the first approach, with rapid judgments taking precedence over extensive deliberation. This may have been influenced by the emotionally charged context and the complexity of genetic testing. In addition, the presence of dementia likely played a pivotal role, as defaulting to the intuitive process may have been a necessary adaptation to cognitive impairment. Family members, in turn, might have prioritized expediency or emotional considerations over a detailed evaluation of potential outcomes. These findings underscore the importance of tailored communication in genetic counseling. By presenting information clearly and accessibly, healthcare professionals can help patients and families make decisions that are not only intuitive but also deliberate.

In our study, patients’ decisions to pursue genetic testing for monogenic causes of dementia were primarily driven by the desire to obtain clarity and actionable information for their relatives, or avoid emotional burden and disruptive impact on their family. As this represents an emerging area of investigation within the broader field of genetic testing, we identified only one study that parallels our investigation. This survey on motivations for genetic testing in patients with cognitive impairment reported similar motivations, focused on benefits for family, research and themselves respectively.^52^ Furthermore, our findings are corroborated by previous evidence in other neurodegenerative disorders demonstrating that genetic testing for ALS and PD is considered valuable for patients and their families in making informed decisions, as well as understanding, treating, and potentially preventing the disease.^37^, ^38^ However, while reporting similar concerns about the emotional burden on patients and their families, individuals with ALS or PD expressed greater worry about financial implications, including the cost of testing, and insurance or employment discrimination. This difference may be attributed to local and legal disparities: in the United States, where these studies were conducted, genetic testing tends to be more costly, and legal protections against genetic discrimination are less comprehensive, whereas in the Netherlands, testing is mostly covered by insurance, and stronger laws are in place to safeguard against genetic discrimination.

This explorative investigation shows that half of memory clinic patients who met eligibility criteria were interested to pursue genetic testing for monogenic causes, and considered the results to be valuable and actionable for themselves and their families. However, data on the implications remain scarce. As a next step, our forthcoming study will report on the emotional, psychological, and behavioral impact of disclosing test results. These findings will provide a fuller picture of perspectives and experiences of patients and families in the context of genetic testing in dementia care.

### 4.2 Strengths and limitations

We included a large cohort of consecutive patients who underwent a standardized diagnostic work‐up and followed a uniform care path, making our sample representative of a real‐world clinical setting with minimal bias. Conducted at a tertiary referral center specializing in young‐onset dementia and often providing second opinions, our study focused on a patient population for whom genetic testing for monogenic causes is warranted. As a result, the proportion of patients eligible for genetic testing was relatively high, and as such not generalizable to primary or secondary care.

If eligible for genetic testing, patients and their family members were invited to participate in our study. In 26% (36/138) of cases, at least one individual consented to be included. However, the sample size was small, with those who declined genetic testing (6/36, 17%) underrepresented in questionnaires and interviews. When asked about their reasons for not participating, many expressed that they did not consider themselves of scientific interest, precisely because their DNA was not analyzed, or they felt overwhelmed with the dementia diagnosis, disease progression, and burden of care. Despite these challenges, we were able to include some participants who decided against genetic testing, enriching our understanding of the full spectrum of considerations of patients and their families. Future research should aim to achieve a more balanced representation by developing strategies to better engage individuals who choose to not learn whether their condition is familial. Furthermore, tailored counseling could help to discern whether those who refrain from genetic testing are reluctant due to the burden of their current circumstances and need additional support, or if they are genuinely uninterested in exploring this option.

Lastly, as the patients visiting our memory clinic are predominantly young, highly educated, and Caucasian, so was our study sample. This lack of diversity underscores the need for future research to include a broader range of populations living with dementia. Efforts should be made to engage underrepresented groups, including those from various racial and ethnic backgrounds, socioeconomic statuses, and educational levels. Inclusive research practices will not only enhance the generalizability of results but also contribute to the development of equitable and effective interventions and support systems for individuals and families dealing with (familial) dementia.

## 5 CONCLUSION

We found that half of memory clinic patients who qualified for genetic testing for monogenic causes pursued this option. Decisions tended to be influenced less by extensive deliberation of knowledge, which was found to be limited, and more by quick, intuitive judgements. Motivations included providing transparency about heredity, enhancing medical and personal actionability, resolving uncertainty, as well as concerns about the emotional burden and disruptive impact on their family. Given the recent advances in developing disease‐modifying therapies and growing opportunities to participate in clinical trials, it is essential to take their perspectives into account to pave the way for more personalized care for those affected by (familial) dementia.

## CONFLICT OF INTEREST STATEMENT

Jetske van der Schaar is a recipient of the Alzheimer Nederland InterAct grant (#WE.08‐2024‐01) and the ZonMw Impact Explorer Dementie (#10510462310005). She wrote a book for a layman's audience about the personal impact of dominantly inherited AD, for which she received grants or contracts from Aegon Nederland and Alzheimer Nederland and royalties from Uitgeverij Prometheus. Jetske van der Schaar is a member of the advisory board for the National Dementia Strategy of the Dutch Ministry of Health, Welfare and Sport. Research programs of Wiesje van der Flier have been funded by ZonMW, NWO, EU‐JPND, EU‐IHI, Alzheimer Nederland, Hersenstichting CardioVascular Onderzoek Nederland, Health∼Holland, Topsector Life Sciences & Health, stichting Dioraphte, Gieskes‐Strijbis fonds, stichting Equilibrio, Edwin Bouw fonds, Pasman stichting, stichting Alzheimer & Neuropsychiatrie Foundation, Philips, Biogen MA Inc, Novartis‐NL, Life‐MI, AVID, Roche BV, Fujifilm, Eisai, Combinostics. WF holds the Pasman chair. WF has been an invited speaker at Biogen MA Inc, Danone, Eisai, WebMD Neurology (Medscape), NovoNordisk, Springer Healthcare, European Brain Council. All funding is paid to her institution. Wiesje van der Flier is a consultant to Oxford Health Policy Forum CIC, Roche, Biogen MA Inc, and Eisai. She is a member of steering committee of NovoNordisk evoke/evoke+. All funding is paid to her institution. WF participated in advisory boards of Biogen MA Inc, Roche, and Eli Lilly. Wiesje van der Flier is member of the steering committee of PAVE and Think Brain Health. She was associate editor of Alzheimer, Research & Therapy in 2020/2021. She is an associate editor at Brain. Leonie Visser has been an invited speaker at an event organized by the Schwabe Group, fees were paid to her institution. Her research has been funded by ZonMW, Alzheimer Nederland, Health∼Holland Topsector Life Sciences & Health, Eisai, and Amsterdam Public Health research institute. None of the other authors have conflicts of interest to disclose. Author disclosures are available in the supporting information.

## Supporting information



Supporting Information

Supporting Information

Supporting Information

Supporting Information

Supporting Information
