# Supplementary material for: Deciding on genetic testing for familial dementia: Perspectives of patients and families
Source: Alzheimers Dement. 2025 Apr 6;21(4):e70140. doi: 10.1002/alz.70140 (PMC11972981; doi:10.1002/alz.70140)
Supplement: Supplementary file 1 — Supporting Information [file ALZ-21-e70140-s001.docx]

**Supplement 1**

T0-questionnaire

During their first visit, participants self-reported various psychosocial characteristics by filling out a digital questionnaire, assessing:

- *perceived susceptibility, perceived severity,* and *experience with dementia* by indicating their level of agreement (on a Likert scale from strongly disagree [1] to strongly agree [5]) with the first two subscales and an added third of the Motivation to Change Lifestyle and Health Behaviors for Dementia Risk Reduction scale (MCLHB-DRR, range: 3-15, 5-25 and 2-10, e.g. “It is very likely that I will be diagnosed with dementia”, “The thought of dementia frightens me”, and “I have been extensively involved in caring for someone with dementia” respectively),^1, 2^
- *perceived likelihood of a genetic cause* for their symptoms with a visual analogue scale (VAS, range: 0-100%),
- *openness to discuss symptoms in the family* by indicating their level of agreement (on a Likert scale from strongly disagree [1] to strongly agree [5]) with an adapted 9-item Openness to Discuss Cancer in the Family scale (ODCF, range: 9-45; e.g., “I talk as little as possible about my symptoms because I don't want to worry my family”)^3^,
- *perceived social support* with the 12-item Multidimensional Scale of Perceived Social Support (MSPSS, range: 12-84),^4, 5^
- *coping strategies* with the 28-item Brief COPE scale (subscales: problem-focused, emotion-focused and avoidant coping; range: 1-4),^6^
- a*nxiety and depression* with the Hospital Anxiety and Depression Scale (HADS; range: 0-21),^7^
- and *quality of life* with the Brunnsviken Brief Quality of Life Scale (BBQ; range: 0-96),^8^ as well as a single Visual Analog Scale (VAS; range: 0-10).

T1-questionnaire

After having been offered DNA testing, participants filled out a digital questionnaire, assessing:

- *beliefs about dementia* by indicating their level of agreement (on a Likert scale from strongly disagree [1] to strongly agree [5]) with 6 statements (range: 0-0; e.g. “Dementia is a normal side effect of aging”),
- *knowledge about monogenic causes of dementia* by evaluating 6 statements as true or false (e.g., “It is rare for dementia to have a hereditary cause”),
- *knowledge of heredity risks* by assigning a probability (on a visual analogue scale, range: 0-100%) to six scenarios (e.g., “If someone has a hereditary cause for dementia, what is the chance for a brother, sister or child to also carry it?”),
- *considerations to (not) be tested* by indicating their level of agreement (on a Likert scale from strongly disagree [1] to strongly agree [5]) with 23 statements (e.g., “If a hereditary cause for my symptoms is found, I could deal with it”)
- and *shared decision-making* by indicating their level of agreement (on a Likert scale from strongly disagree [1] to strongly agree [5]) with 11 statements (e.g., “My family’s opinion influences my decision whether or not to consent to DNA testing”).

All scales were self-constructed, drawing from literature on factors potentially associated with interest in and impact of DNA testing for genetically inherited disorders.

**References**

1. Kim S, Sargent-Cox K, Cherbuin N, Anstey KJ. Development of the motivation to change lifestyle and health behaviours for dementia risk reduction scale. *Dement Geriatr Cogn Dis Extra*. May 2014;4(2):172-83. doi:10.1159/000362228

2. Joxhorst T, Vrijsen J, Niebuur J, Smidt N. Cross-cultural validation of the motivation to change lifestyle and health behaviours for dementia risk reduction scale in the Dutch general population. *BMC Public Health*. 2020/07/20 2020;20(1):788. doi:10.1186/s12889-020-08737-y

3. Mesters I, van den Borne H, McCormick L, Pruyn J, de Boer M, Imbos T. Openness to discuss cancer in the nuclear family: scale, development, and validation. *Psychosom Med*. May-Jun 1997;59(3):269-79. doi:10.1097/00006842-199705000-00010

4. Zimet GD, Powell SS, Farley GK, Werkman S, Berkoff KA. Psychometric characteristics of the Multidimensional Scale of Perceived Social Support. *J Pers Assess*. Winter 1990;55(3-4):610-7. doi:10.1080/00223891.1990.9674095

5. van Oostrom I, Meijers-Heijboer H, Lodder LN, et al. Long-term psychological impact of carrying a BRCA1/2 mutation and prophylactic surgery: a 5-year follow-up study. *J Clin Oncol*. Oct 15 2003;21(20):3867-74. doi:10.1200/jco.2003.10.100

6. Carver CS. You want to measure coping but your protocol's too long: consider the brief COPE. *Int J Behav Med*. 1997;4(1):92-100. doi:10.1207/s15327558ijbm0401_6

7. Zigmond AS, Snaith RP. The hospital anxiety and depression scale. *Acta Psychiatr Scand*. Jun 1983;67(6):361-70. doi:10.1111/j.1600-0447.1983.tb09716.x

8. Lindner P, Frykheden O, Forsström D, et al. The Brunnsviken Brief Quality of Life Scale (BBQ): Development and Psychometric Evaluation. *Cogn Behav Ther*. Apr 2016;45(3):182-95. doi:10.1080/16506073.2016.1143526
