# Supplementary material for: Deciding on genetic testing for familial dementia: Perspectives of patients and families
Source: Alzheimers Dement. 2025 Apr 6;21(4):e70140. doi: 10.1002/alz.70140 (PMC11972981; doi:10.1002/alz.70140)
Supplement: Supplementary file 2 — Supporting Information [file ALZ-21-e70140-s002.docx]

**Supplement 2**


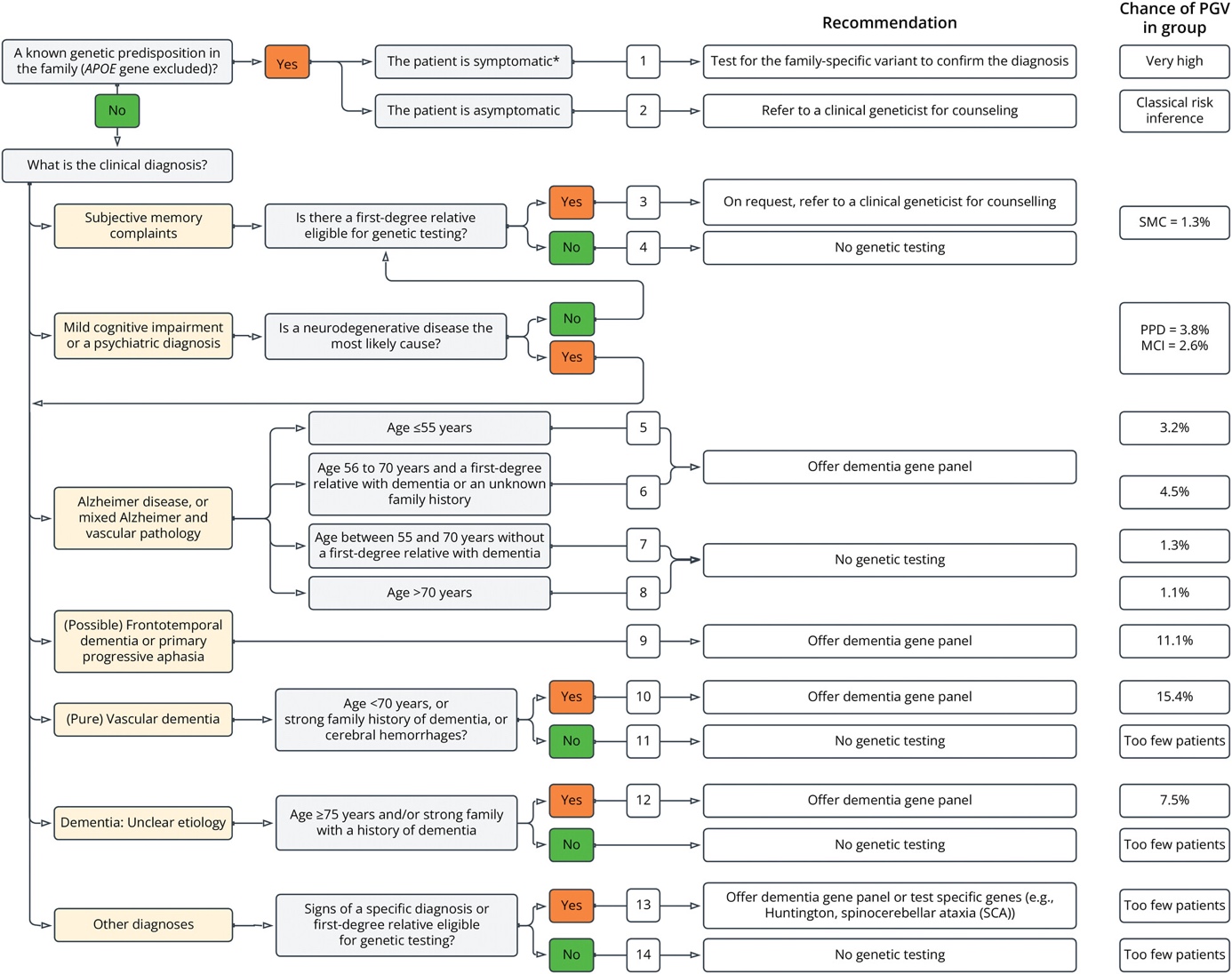


*Figure 2 Criteria to Determine Eligibility for Genetic Testing in a Memory Clinic Setting (Reproduced from Van Der Lee et al.[1], licensed under CC BY 4.0.)*

Eligibility is based on clinical diagnosis, age of presentation and family history in first-degree relatives. The dementia gene panel consists of 54 dementia related genes, the *APP* duplication, and the *C9ORF72* repeat expansion.

* as determined by neuropsychological investigation

PGV, pathogenic genetic variant.

**References**

[1] Van Der Lee SJ, Hulsman M, Van Spaendonk R, Van Der Schaar J, Dijkstra J, Tesi N, et al. Prevalence of Pathogenic Variants and Eligibility Criteria for Genetic Testing in Patients Who Visit a Memory Clinic. Neurology. 2025;104:e210273.
