# Supplementary material for: Deciding on genetic testing for familial dementia: Perspectives of patients and families
Source: Alzheimers Dement. 2025 Apr 6;21(4):e70140. doi: 10.1002/alz.70140 (PMC11972981; doi:10.1002/alz.70140)
Supplement: Supplementary file 4 — Supporting Information [file ALZ-21-e70140-s005.docx]

**Supplement 4**

T1-questionnaire

Table 1 Beliefs about dementia

|  |  | **DNA tested** | |  |
| --- | --- | --- | --- | --- |
| **Statement** | **all (n (%))**  ***n* = 33** | **no (n (%))**  ***n* = 6** | **yes (n %))**  ***n* = 27** | ***p*-value** |
| Dementia is a normal side effect of aging. |  |  |  | 0.700 |
| Strongly disagree | 6 (18%) | 1 (17%) | 5 (19%) |  |
| Disagree | 10 (30%) | 1 (17%) | 9 (33%) |  |
| Neutral | 11 (33%) | 3 (50%) | 8 (30%) |  |
| Agree | 4 (12%) | 1 (17%) | 3 (11%) |  |
| Strongly agree | 2 (6%) |  | 2 (7%) |  |
| Dementia results from biological changes in the brain. |  |  |  | 0.210 |
| Strongly disagree | 0 (0%) | 0 (0%) | 0 (0%) |  |
| Disagree | 2 (6%) | 0 (0%) | 2 (7%) |  |
| Neutral | 12 (36%) | 4 (67%) | 8 (30%) |  |
| Agree | 13 (39%) | 2 (33%) | 11 (41%) |  |
| Strongly agree | 6 (18%) | 0 (0%) | 6 (22%) |  |
| The development dementia can be prevented or slowed with a healthy lifestyle. |  |  |  | 0.660 |
| Strongly disagree | 2 (6%) | 0 | 2 (7%) |  |
| Disagree | 4 (12%) | 1 (17%) | 3 (11%) |  |
| Neutral | 10 (30%) | 3 (50%) | 7 (26%) |  |
| Agree | 12 (36%) | 1 (17%) | 11 (41%) |  |
| Strongly agree | 5 (15%) | 1 (17%) | 4 (15%) |  |
| Symptoms of dementia can be somewhat suppressed with medication. |  |  |  | 0.940 |
| Strongly disagree | 4 (12%) | 0 (0%) | 4 (15%) |  |
| Disagree | 5 (15%) | 2 (33%) | 3 (11%) |  |
| Neutral | 13 (39%) | 2 (33%) | 11 (41%) |  |
| Agree | 9 (27%) | 2 (33%) | 7 (26%) |  |
| Strongly agree | 2 (6%) | 0 (0%) | 2 (7%) |  |
| The development of dementia can be slowed with treatment. |  |  |  | 0.940 |
| Strongly disagree | 6 (18%) | 1 (17%) | 5 (19%) |  |
| Disagree | 3 (9%) | 0 (0%) | 3 (11%) |  |
| Neutral | 10 (30%) | 3 (50%) | 7 (26%) |  |
| Agree | 14 (42%) | 2 (33%) | 12 (44%) |  |
| Strongly agree | 0 (0%) | 0 (0%) | 0 (0%) |  |
| For the next generation, treatments to prevent dementia will be available. |  |  |  | 0.610 |
| Strongly disagree | 2 (6%) | 0 (0%) | 2 (7%) |  |
| Disagree | 2 (6%) | 0 (0%) | 2 (7%) |  |
| Neutral | 18 (55%) | 4 (67%) | 14 (52%) |  |
| Agree | 8 (24%) | 1 (17%) | 7 (26%) |  |
| Strongly agree | 3 (9%) | 1 (17%) | 2 (7%) |  |

Table 2 Knowledge about monogenic causes of dementia

|  |  | **DNA tested** | |  |
| --- | --- | --- | --- | --- |
| **Statement** | **all (n (%))**  ***n* = 33** | **no (n (%))**  ***n* = 6** | **yes (n (%))**  ***n* = 27** | ***p*-value** |
| It is rare for dementia to have a hereditary cause. |  |  |  | 0.320 |
| True | 16 (48%) | 4 (67%) | 12 (44%) |  |
| False | 17 (52%) | 2 (33%) | 15 (56%) |  |
| With a hereditary cause, symptoms often begin after age 65. |  |  |  | 0.680 |
| True | 14 (42%) | 3 (50%) | 11 (41%) |  |
| False | 19 (58%) | 3 (50%) | 16 (59%) |  |
| If someone has a hereditary cause for dementia, they will almost certainly become ill. |  |  |  | 0.120 |
| True | 15 (45%) | 1 (17%) | 14 (52%) |  |
| False | 18 (55%) | 5 (83%) | 13 (48%) |  |
| A hereditary cause for dementia can lead to higher premiums when acquiring insurance. |  |  |  | 0.710 |
| True | 9 (27%) | 2 (33%) | 7 (26%) |  |
| False | 9 (73%) | 4 (67%) | 20 (74%) |  |
| It is possible to prevent a hereditary cause for dementia from being passed on to a child. |  |  |  | 0.210 |
| True | 13 (39%) | 1 (17%) | 12 (44%) |  |
| False | 20 (61%) | 5 (83%) | 15 (56%) |  |
| If the disease has a hereditary cause for dementia, it can always be found with genetic testing. |  |  |  | 0.180 |
| True | 19 (58%) | 2 (33%) | 17 (63%) |  |
| False | 14 (42) | 4 (67%) | 10 (37%) |  |

Table 3 Knowledge of heredity risks

|  |  | **DNA tested** | |  |
| --- | --- | --- | --- | --- |
| **Statement** | **all (%±sd)**  ***n* = 33** | **no (%±sd)**  ***n* = 6** | **yes (%±sd)**  ***n* = 27** | ***p*-value** |
| What is the average risk of dementia in The Netherlands? | 36±18 | 37±18 | 36±18 | 0.970 |
| If someone has a hereditary cause for dementia, what is his or her risk of dementia? | 49±27 | 39±22 | 51±28 | 0.340 |
| If someone has a hereditary cause for dementia, what is the likelihood that a sibling or child will also have it? | 43±24 | 36±19 | 44±25 | 0.470 |
| If a sibling or child has an inherited cause for dementia, what is his or her risk of developing dementia? | 45±28 | 34±20 | 48±29 | 0.270 |
| If a sibling or child does not have the hereditary cause for dementia, what is the risk that he or she will develop dementia? | 24±20 | 20±23 | 25±20 | 0.560 |
| How would you assess the likelihood of a hereditary cause for your symptoms? | 34±33 | 9±11 | 39±33 | 0.040 * |

Table 4 Considerations to (not) be tested

|  |  | **DNA tested** | |  |
| --- | --- | --- | --- | --- |
| **Statement** | **all (n (%))**  ***n* = 33** | **no (n (%))**  ***n* = 6** | **yes (n %))**  ***n* = 27** | ***p*-value** |
| I don't want to know the cause of my symptoms, because there is little I can do about it anyway. |  |  |  | 0.720 |
| Strongly disagree | 22 (67%) | 4 (67%) | 18 (67%) |  |
| Disagree | 7 (21%) |  | 7 (26%) |  |
| Neutral | 2 (6%) | 1 (17%) | 1 (4%) |  |
| Agree | 2 (6%) | 1 (17%) | 1 (4%) |  |
| Strongly agree |  |  |  |  |
| I struggle with the uncertainty of not knowing the cause of my symptoms, and prefer to know where I stand. |  |  |  | 0.360 |
| Strongly disagree | 2 (6%) |  | 2 (7%) |  |
| Disagree | 2 (6%) | 2 (33%) |  |  |
| Neutral | 5 (15%) | 1 (17%) | 4 (15%) |  |
| Agree | 11 (33%) | 1 (17%) | 10 (37%) |  |
| Strongly agree | 13 (39%) | 2 (33%) | 11 (41%) |  |
| I want to know the cause of my symptoms so that I can receive better care and support. |  |  |  | 0.100 |
| Strongly disagree |  |  |  |  |
| Disagree | 2 (6%) | 1 (17%) | 1 (4%) |  |
| Neutral | 3 (9%) | 1 (17%) | 2 (7%) |  |
| Agree | 14 (42%) | 3 (50%) | 11 (41%) |  |
| Strongly agree | 14 (42%) | 1 (17%) | 13 (48%) |  |
| With the results of the test, I can better prepare for the future (for example, by arranging care, advanced directives and will). |  |  |  | 0.180 |
| Strongly disagree |  |  |  |  |
| Disagree | 1 (3%) | 1 (17%) |  |  |
| Neutral | 4 (12%) | 2 (33%) | 2 (7%) |  |
| Agree | 15 (45%) | 1 (17%) | 14 (52%) |  |
| Strongly agree | 13 (39%) | 2 (33%) | 11 (41%) |  |
| Based on the results of the test, I would change my lifestyle. |  |  |  | 0.920 |
| Strongly disagree | 1 (3%) |  | 1 (4%) |  |
| Disagree | 7 (21%) | 2 (33%) | 5 (19%) |  |
| Neutral | 11 (33%) | 1 (17%) | 10 (37%) |  |
| Agree | 10 (30%) | 2 (33%) | 8 (30%) |  |
| Strongly agree | 4 (12%) | 1 (17%) | 3 (11%) |  |
| If a hereditary cause for my symptoms is found, I would be able to cope with it. |  |  |  | 0.080 |
| Strongly disagree |  |  |  |  |
| Disagree | 2 (6%) | 2 (33%) |  |  |
| Neutral | 9 (27%) | 2 (33%) | 7 (26%) |  |
| Agree | 15 (45%) | 1 (17%) | 14 (52%) |  |
| Strongly agree | 7 (21%) | 1 (17%) | 6 (22%) |  |
| If a hereditary cause for my symptoms is found, life would lose its meaning to me. |  |  |  | 0.270 |
| Strongly disagree | 11 (33%) | 1 (17%) | 10 (37%) |  |
| Disagree | 15 (45%) | 3 (50%) | 12 (44%) |  |
| Neutral | 5 (15%) | 1 (17%) | 4 (15%) |  |
| Agree | 2 (6%) | 1 (17%) | 1 (4%) |  |
| Strongly agree |  |  |  |  |
| I think my family should be aware of information about heredity. |  |  |  | 0.080 |
| Strongly disagree |  |  |  |  |
| Disagree | 1 (3%) |  | 1 (4%) |  |
| Neutral | 3 (9%) | 3 (50%) |  |  |
| Agree | 11 (33%) | 1 (17%) | 10 (37%) |  |
| Strongly agree | 18 (55%) | 2 (33%) | 16 (59%) |  |
| I want to know the risk of dementia for my siblings or children. |  |  |  | 0.030 * |
| Strongly disagree |  |  |  |  |
| Disagree |  |  |  |  |
| Neutral | 4 (12%) | 3 (50%) | 1 (4%) |  |
| Agree | 16 (48%) | 2 (33%) | 14 (52%) |  |
| Strongly agree | 13 (39%) | 1 (17%) | 12 (44%) |  |
| If a hereditary cause is found, I would worry about my family. |  |  |  | 0.770 |
| Strongly disagree | 2 (6%) |  | 2 (7%) |  |
| Disagree | 1 (3%) |  | 1 (4%) |  |
| Neutral | 8 (24%) | 1 (17%) | 7 (26%) |  |
| Agree | 13 (39%) | 4 (67%) | 9 (33%) |  |
| Strongly agree | 9 (27%) | 1 (17%) | 8 (30%) |  |
| I don't want to burden my family with information about heredity. |  |  |  | 0.360 |
| Strongly disagree | 6 (18%) | 1 (17%) | 5 (19%) |  |
| Disagree | 14 (42%) | 1 (17%) | 13 (48%) |  |
| Neutral | 11 (33%) | 4 (67%) | 7 (26%) |  |
| Agree | 1 (3%) |  | 1 (4%) |  |
| Strongly agree | 1 (3%) |  | 1 (4%) |  |
| If a hereditary cause is found I would feel guilty that my siblings or children might also have the hereditary cause. |  |  |  | 0.280 |
| Strongly disagree | 9 (27%) |  | 9 (33%) |  |
| Disagree | 10 (30%) | 2 (33%) | 8 (30%) |  |
| Neutral | 9 (27%) | 4 (67%) | 5 (19%) |  |
| Agree | 3 (9%) |  | 3 (11%) |  |
| Strongly agree | 2 (6%) |  | 2 (7%) |  |
| If a hereditary cause is found, my family can prevent the hereditary cause from being passed on to their children. |  |  |  | 0.810 |
| Strongly disagree | 1 (3%) | 1 (17%) |  |  |
| Disagree | 8 (24%) | 2 (33%) | 6 (22%) |  |
| Neutral | 11 (33%) |  | 11 (41%) |  |
| Agree | 11 (33%) | 2 (33%) | 9 (33%) |  |
| Strongly agree | 2 (6%) | 1 (17%) | 1 (4%) |  |
| The results of DNA testing can help my family members make informed choices for the future. |  |  |  | 0.210 |
| Strongly disagree |  |  |  |  |
| Disagree | 1 (3%) | 1 (17%) |  |  |
| Neutral | 7 (21%) | 2 (33%) | 5 (19%) |  |
| Agree | 19 (58%) | 2 (33%) | 17 (63%) |  |
| Strongly agree | 6 (18%) | 1 (17%) | 5 (19%) |  |
| If a hereditary cause is found, it would deteriorate relationships in my family. |  |  |  | 0.030 * |
| Strongly disagree | 13 (39%) |  | 13 (48%) |  |
| Disagree | 12 (36%) | 3 (50%) | 9 (33%) |  |
| Neutral | 3 (9%) | 1 (17%) | 2 (7%) |  |
| Agree | 5 (15%) | 2 (33%) | 3 (11%) |  |
| Strongly agree |  |  |  |  |
| With a hereditary cause, I would receive more understanding and support from people around me. |  |  |  | 1.000 |
| Strongly disagree | 3 (9%) |  | 3 (11%) |  |
| Disagree | 9 (27%) | 3 (50%) | 6 (22%) |  |
| Neutral | 12 (36%) | 1 (17%) | 11 (41%) |  |
| Agree | 6 (18%) | 1 (17%) | 5 (19%) |  |
| Strongly agree | 3 (9%) | 1 (17%) | 2 (7%) |  |
| Others would avoid me if they knew that the dementia in my family is hereditary. |  |  |  | 0.940 |
| Strongly disagree | 13 (39%) | 2 (33%) | 11 (41%) |  |
| Disagree | 13 (39%) | 3 (50%) | 10 (37%) |  |
| Neutral | 7 (21%) | 1 (17%) | 6 (22%) |  |
| Agree |  |  |  |  |
| Strongly agree |  |  |  |  |
| I worry that the results of DNA testing will be shared with third parties, such as insurers or employers. |  |  |  | 0.400 |
| Strongly disagree | 2 (6%) |  | 2 (7%) |  |
| Disagree | 12 (36%) | 2 (33%) | 10 (37%) |  |
| Neutral | 5 (15%) | 1 (17%) | 4 (15%) |  |
| Agree | 9 (27%) | 1 (17%) | 8 (30%) |  |
| Strongly agree | 5 (15%) | 2 (33%) | 3 (11%) |  |
| I worry that a hereditary cause will lead to higher insurance premiums for my family. |  |  |  | 0.280 |
| Strongly disagree | 4 (12%) |  | 4 (15%) |  |
| Disagree | 8 (24%) | 2 (33%) | 6 (22%) |  |
| Neutral | 10 (30%) | 1 (17%) | 9 (33%) |  |
| Agree | 8 (24%) | 1 (17%) | 7 (26%) |  |
| Strongly agree | 3 (9%) | 2 (33%) | 1 (4%) |  |
| I am concerned that an inherited cause will lead to discrimination against my family, for example in their work. |  |  |  | 0.710 |
| Strongly disagree | 5 (15%) | 1 (17%) | 4 (15%) |  |
| Disagree | 15 (45%) | 2 (33%) | 13 (48%) |  |
| Neutral | 7 (21%) | 2 (33%) | 5 (19%) |  |
| Agree | 5 (15%) |  | 5 (19%) |  |
| Strongly agree | 1 (3%) | 1 (17%) |  |  |
| I am concerned about the costs of DNA testing. |  |  |  | 0.590 |
| Strongly disagree | 6 (18%) | 1 (17%) | 5 (19%) |  |
| Disagree | 13 (39%) | 3 (50%) | 10 (37%) |  |
| Neutral | 9 (27%) | 2 (33%) | 7 (26%) |  |
| Agree | 3 (9%) |  | 3 (11%) |  |
| Strongly agree | 2 (6%) |  | 2 (7%) |  |
| If a hereditary cause for my symptoms is found, I would like to participate in scientific research. |  |  |  | 0.110 |
| Strongly disagree |  |  |  |  |
| Disagree | 2 (6%) | 2 (33%) |  |  |
| Neutral | 10 (30%) | 1 (17%) | 9 (33%) |  |
| Agree | 14 (42%) | 3 (50%) | 11 (41%) |  |
| Strongly agree | 7 (21%) |  | 7 (26%) |  |
| This is a good time for me to undergo DNA testing. |  |  |  | 0.030 * |
| Strongly disagree | 1 (3%) | 1 (17%) |  |  |
| Disagree | 2 (6%) | 2 (33%) |  |  |
| Neutral | 6 (18%) |  | 6 (22%) |  |
| Agree | 15 (45%) | 3 (50%) | 12 (44%) |  |
| Strongly agree | 9 (27%) |  | 9 (33%) |  |

Table 5 Social influences on decision-making

|  |  | **DNA tested** | |  |
| --- | --- | --- | --- | --- |
| **Statement** | **all (n (%))**  ***n* = 33** | **no (n (%))**  ***n* = 6** | **yes (n %))**  ***n* = 27** | ***p*-value** |
| My doctor's opinion influences my decision whether or not to consent to DNA testing. |  |  |  | 0.530 |
| Strongly disagree | 7 (22%) | 1 (17%) | 6 (23%) |  |
| Disagree | 9 (28%) | 2 (33%) | 7 (27%) |  |
| Neutral | 9 (28%) | 1 (17%) | 8 (31%) |  |
| Agree | 5 (16%) |  | 5 (19%) |  |
| Strongly agree | 2 (6%) | 2 (33%) |  |  |
| I have discussed the possibility of DNA testing with my partner. |  |  |  | 0.020 * |
| Strongly disagree | 1 (3%) |  | 1 (4%) |  |
| Disagree | 1 (3%) |  | 1 (4%) |  |
| Neutral | 6 (19%) |  | 6 (23%) |  |
| Agree | 11 (34%) | 1 (17%) | 10 (38%) |  |
| Strongly agree | 13 (41%) | 5 (83%) | 8 (31%) |  |
| I have found support from my partner for my considerations regarding DNA testing. |  |  |  | 0.090 |
| Strongly disagree |  |  |  |  |
| Disagree | 1 (3%) |  | 1 (4%) |  |
| Neutral | 8 (25%) |  | 8 (31%) |  |
| Agree | 10 (31%) | 2 (33%) | 8 (31%) |  |
| Strongly agree | 13 (41%) | 4 (67%) | 9 (35%) |  |
| My partner's opinion has influenced my decision whether or not to consent to DNA testing. |  |  |  | 0.640 |
| Strongly disagree | 3 (9%) | 1 (17%) | 2 (8%) |  |
| Disagree | 6 (19%) | 1 (17%) | 5 (19%) |  |
| Neutral | 10 (31%) |  | 10 (38%) |  |
| Agree | 8 (25%) | 3 (50%) | 5 (19%) |  |
| Strongly agree | 5 (16%) | 1 (17%) | 4 (15%) |  |
| I have discussed the possibility of DNA testing with my family. |  |  |  | 0.870 |
| Strongly disagree | 1 (3%) | 1 (17%) |  |  |
| Disagree | 1 (3%) |  | 1 (4%) |  |
| Neutral | 3 (9%) |  | 3 (12%) |  |
| Agree | 16 (50%) | 3 (50%) | 13 (50%) |  |
| Strongly agree | 11 (34%) | 2 (33%) | 9 (35%) |  |
| I have found support from my family for my considerations regarding DNA testing. |  |  |  | 0.400 |
| Strongly disagree |  |  |  |  |
| Disagree | 1 (3%) |  | 1 (4%) |  |
| Neutral | 7 (22%) | 1 (17%) | 6 (23%) |  |
| Agree | 13 (41%) | 2 (33%) | 11 (42%) |  |
| Strongly agree | 11 (34%) | 3 (50%) | 8 (31%) |  |
| My family's opinion influences my decision whether or not to consent to DNA testing. |  |  |  | 0.010 * |
| Strongly disagree | 5 (16%) |  | 5 (19%) |  |
| Disagree | 5 (16%) |  | 5 (19%) |  |
| Neutral | 8 (25%) | 1 (17%) | 7 (27%) |  |
| Agree | 12 (38%) | 3 (50%) | 9 (35%) |  |
| Strongly agree | 2 (6%) | 2 (33%) |  |  |
| I have talked to others about the possibility of DNA testing. |  |  |  | 0.300 |
| Strongly disagree | 6 (19%) | 2 (33%) | 4 (15%) |  |
| Disagree | 5 (16%) | 1 (17%) | 4 (15%) |  |
| Neutral | 12 (38%) | 2 (33%) | 10 (38%) |  |
| Agree | 7 (22%) | 1 (17%) | 6 (23%) |  |
| Strongly agree | 2 (6%) |  | 2 (8%) |  |
| I have found support from others for my considerations regarding DNA testing. |  |  |  | 0.180 |
| Strongly disagree | 4 (12%) | 2 (33%) | 2 (8%) |  |
| Disagree | 4 (12%) | 1 (17%) | 3 (12%) |  |
| Neutral | 16 (50%) | 2 (33%) | 14 (54%) |  |
| Agree | 8 (25%) | 1 (17%) | 7 (27%) |  |
| Strongly agree |  |  |  |  |
| The opinions of others influence my decision whether or not to consent to DNA testing. |  |  |  | 0.700 |
| Strongly disagree | 8 (25%) | 2 (33%) | 6 (23%) |  |
| Disagree | 10 (31%) | 2 (33%) | 8 (31%) |  |
| Neutral | 11 (34%) | 1 (17%) | 10 (38%) |  |
| Agree | 3 (9%) | 1 (17%) | 2 (8%) |  |
| Strongly agree |  |  |  |  |
| I feel that I am free to choose whether or not to consent to DNA testing without pressure from others. |  |  |  | 0.380 |
| Strongly disagree | 1 (3%) |  | 1 (4%) |  |
| Disagree |  |  |  |  |
| Neutral | 3 (9%) |  | 3 (12%) |  |
| Agree | 11 (34%) | 2 (33%) | 9 (35%) |  |
| Strongly agree | 17 (53%) | 4 (67%) | 13 (50%) |  |
